# Systematic chemical screening identifies disulfiram as a repurposed drug that enhances sensitivity to cisplatin in bladder cancer: a summary of preclinical studies

**DOI:** 10.1038/s41416-019-0609-0

**Published:** 2019-11-01

**Authors:** Yuki Kita, Akihiro Hamada, Ryoichi Saito, Yuki Teramoto, Ryusuke Tanaka, Keishi Takano, Kenji Nakayama, Kaoru Murakami, Keiyu Matsumoto, Shusuke Akamatsu, Toshinari Yamasaki, Takahiro Inoue, Yasuhiko Tabata, Yasushi Okuno, Osamu Ogawa, Takashi Kobayashi

**Affiliations:** 1 Department of Urology, Kyoto University Graduate School of Medicine, Kyoto, Japan; 2 Department of Diagnostic Pathology, Kyoto University Graduate School of Medicine, Kyoto, Japan; 3 Laboratory of Biomaterials, Department of Regeneration Science and Engineering, Institute for Frontier Life and Medical Sciences, Kyoto University, Kyoto, Japan; 4 Department of Environmental and Health Sciences, Hokkaido Institute of Public Health, Sapporo, Japan; 5 Shimadzu Techno-research, Kyoto, Japan; 6 Department of Clinical System Onco-Informatics, Kyoto University Graduate School of Medicine, Kyoto, Japan

**Keywords:** Drug development, Chemotherapy, Bladder cancer, High-throughput screening

## Abstract

**Background:**

Since the standard gemcitabine and cisplatin (GC) chemotherapy for advanced bladder cancer yields limited therapeutic effect due to chemoresistance, it is a clinical challenge to enhance sensitivity to GC.

**Methods:**

We performed high-throughput screening by using a library of known chemicals and repositionable drugs. A total of 2098 compounds were administered alone or with GC to human bladder cancer cells, and chemicals that enhanced GC effects were screened.

**Results:**

Disulfiram (DSF), an anti-alcoholism drug, was identified as a candidate showing synergistic effects with cisplatin but not with gemcitabine in multiple cell lines. Co-administration of DSF with GC affected cellular localisation of a cisplatin efflux transporter ATP7A, increased DNA–platinum adducts and promoted apoptosis. Micellar DSF nanoparticles (DSF-NP) that stabilised DSF in vivo, enhanced the inhibitory effect of cisplatin in patient-derived and cell-based xenograft models without severe adverse effects. A drug susceptibility evaluation system by using cancer tissue-originated spheroid culture showed promise in identifying cases who would benefit from DSF with cisplatin.

**Conclusions:**

The present study highlighted the advantage of drug repurposing to enhance the efficacy of anticancer chemotherapy. Repurposing of DSF to a chemotherapy sensitiser may provide additional efficacy with less expense by using an available drug with a well-characterised safety profile.

## Background

Bladder cancer is the ninth most common cancer worldwide, with 430,000 new cases and 165,000 deaths worldwide each year.^1^ Muscle-invasive bladder cancer (staging classification T2 and above) is associated with high rates of lymph node involvement and distant metastasis. Metastatic bladder cancer cannot be completely resected by surgery. Thus, muscle-invasive bladder cancer and metastatic bladder cancer have a very poor prognosis, with 5-year survival rates of 50 and 20%, respectively.^2^

Cytotoxic chemotherapy has been the standard treatment for advanced bladder cancer. However, treatment outcomes in unresectable urothelial carcinoma (UC) did not improve for more than 30 years until very recently, with the introduction of immune checkpoint inhibitors. Despite the current trends for the increasing use of immune checkpoint inhibitors, the response rates are far from sufficient.^3^ Thus, platinum-based cytotoxic chemotherapy is still the treatment of choice for advanced bladder cancer in many contexts. However, the initial response rate for gemcitabine (GEM) and cisplatin (CDDP), one of the standard systemic chemotherapies for advanced bladder cancer, is ~50–70% due to intrinsic resistance.^4^ Even in responders, many tumours develop acquired resistance and regrow after a short time. Thus, the development of a novel method to enhance the efficacy of cytotoxic chemotherapy for advanced bladder cancer without compromising tolerability is critical.

In recent years, the concept of drug repurposing, in which existing approved drugs are developed and adapted as a therapeutic drug for other diseases, has attracted attention.^5^ The established safety of the candidate compounds provides several advantages compared with the development of new therapeutic compounds. The time and cost required to advance a candidate treatment into clinical trials can also be substantially reduced because in vitro and in vivo screening, chemical optimisation, toxicology studies, bulk manufacturing and formulation development have, in many cases, already been completed and can therefore be bypassed.

To develop a novel therapeutic strategy that enhances the efficiency of GC chemotherapy for bladder cancer, here we applied a high-throughput screening by using a library of known chemicals and repositionable drugs. We demonstrate a novel strategy with potential clinical application, from in vitro screening, through proof-of-principle experiments, to preclinical evaluation in mice and development of an efficacy prediction system by using cancer tissue-originated spheroid (CTOS) culture of human bladder cancer.

## Methods

### Cell culture

Human bladder cancer cell lines UMUC3, J82, T24, HT1197 and HT1376 were purchased from American Type Culture Collection (Manassas, VA) within 6 months from the initiation of this study. Cells were cultured in RPMI-1640 supplemented with 10% foetal bovine serum, 25 mM HEPES, 100 U/mL penicillin and 100 U/mL streptomycin in a 37 °C humidified environment containing 5% CO_2_.

### Compound library

Screening compounds were provided from the Medical Research Support Center, Graduate School of Medicine, Kyoto University. The library consists of 2098 known drugs and small-molecule compounds with a history of use in human clinical trials, collected from Prestwick Chemical library, Calbiochem® inhibitors and Selleckchem® inhibitor library. The compounds were solubilised in dimethyl sulfoxide (DMSO) to a final concentration of 10 mM, formatted in 96-well microtiter plates and stored at −80 °C.

### Screening methodology

**Fig. 1:**
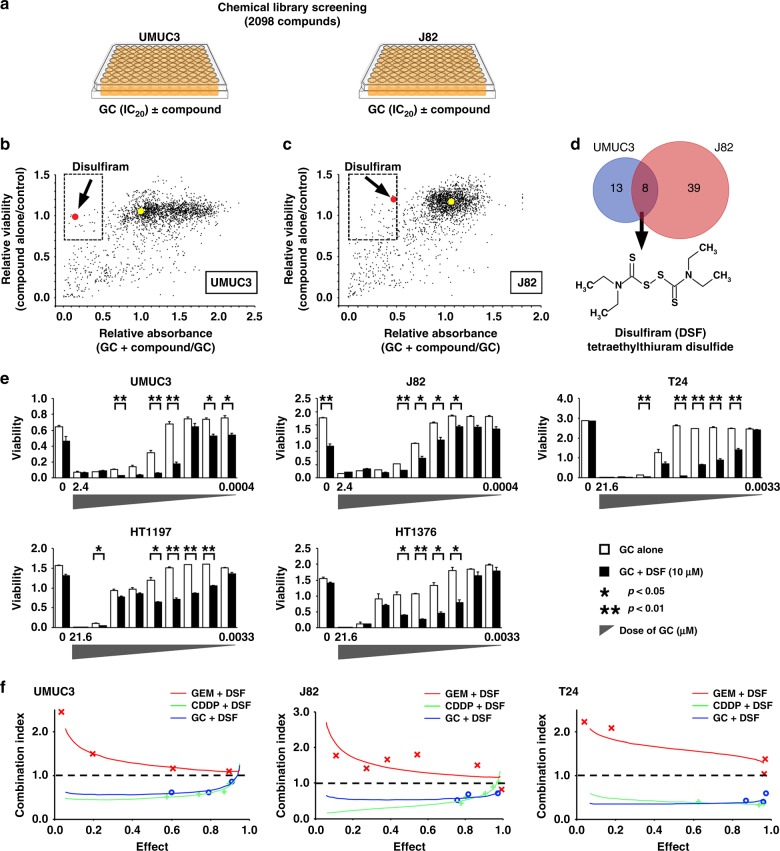
Combinatorial high-throughput screen identified DSF as a synergistic sensitiser of CDDP. **a** Chemical screening strategy. UMUC3 and J82 cells were treated with GC at IC_20_ concentrations alone or in combination with 2,098 compounds from the chemical library. After 72 h, cell viability was determined by using WST-8 assay. **b**, **c** Each compound is expandedly scattered according to the relative cell viability of treatment with compound alone to the control (water) and relative cell viability of treatment with GC plus compound to GC alone (**b**; UMUC3, **c**; J82). Hit compounds are shown in the dashed squares (compound alone/control ≥ 0.7 and GC + compound/GC ≤ 0.5). Red dots indicate DSF and yellow dots indicate vehicle. **d** Venn diagram showing eight compounds that sensitised both UMUC3 and J82 cells to GC in the initial screening. Further validation narrowed the compounds into the strongest sensitiser, DSF, an FDA-approved drug for alcoholism. **e** Enhancement of GC cytotoxicity by DSF in bladder cancer cells. Five bladder cancer cell lines were treated with 10 μM DSF, and varied concentrations of GEM (indicated on the *x* axis) and CDDP were fixed at a ratio of 1:100 for 72 h and evaluated by WST-8 assay. **f** Combination index for combined treatments of Gem plus DSF, CDDP plus DSF and GC plus DSF was determined by using CalcuSyn software. A combination index < 1 (dotted line) denotes synergy. DSF shows synergism with CDDP but not with Gem in all cell lines

All 2098 compounds were tested in UMUC3 and J82 cells in duplicate. Cells were seeded in 96-well tissue culture plates. The following day, each of the compounds was added to a well at a final concentration of 10 µM with or without GEM and CDDP (fixed ratio of 1:100) (Fig. 1a). The fixed ratio of cisplatin to gemcitabine was determined according to a previous study^6^ that adopted the ratio based on the respective IC_50s_ for gemcitabine and cisplatin. We confirmed this by ourselves for the two cell lines we used in the initial screening: 1:87.7 for UMUC3 and 1:63.9 for J82. Then we determined IC_20_ values of concentrations of gemcitabine and cisplatin cocktail (1:100) for each cell line. Each plate had a column of untreated wells for normalisation between plates. After 72 h, water-soluble tetrazolium salt-8 (WST-8) was added, plates were read at 450 nm by a plate reader and statistical computation was done. The optical density (OD) 450 for a compound-treated well was normalised by dividing with the average of the untreated wells on the same plate. Hit compounds were selected with [GC + compound/GC < 0.5] and [compound alone/vehicle > 0.7] in both cell lines.

### Western blot

Cell lysis, sodium dodecyl sulfate–polyacrylamide gel electrophoresis and Western blot analysis was performed as described previously.^7^

### In vitro cytotoxicity assays and dose-effect analysis

Cells plated in 96-well plates were treated with 10 µM DSF and various concentrations of GEM and CDDP with a fixed ratio of 1:100 for 72 h and assayed by WST-8 assay. Combination index values for the treatments were determined by using CalcuSyn software (Biosoft, Ferguson, MO, USA). Combination index values of <1, =1 and >1 indicated synergism, additive and antagonism between the drugs, respectively.

### Apoptosis assay

Apoptosis was determined by using the FITC-conjugated Annexin-V/PI assay kit (Roche, Basel, Switzerland) and flow cytometry following the manufacturer's instructions. Annexin-V-positive cells were classified as early apoptotic cells, and Annexin V and PI double-stained cells were classified as late apoptotic or necrotic cells.

### Quantitative analysis of intracellular reactive oxygen species (ROS) and platinum (Pt)–DNA adducts

Production of ROS was analysed by flow cytometry, as described previously.^8^ For intracellular Pt–DNA adducts, UMUC3 cells were treated with 10 μM DSF and GC (CDDP, 100 µM; GEM, 10 nM) for 1 h, and DNA was extracted by using the QIAampDNA MiniKit (Qiagen, Hilden, Germany). Extracted DNA was resuspended in 0.5 mL of nitric acid and digested with 10 mL of nitric acid and 0.1 mL of perchloric acid for 3 h at 180 °C. The samples were fixed in 3 mL of 0.1 M nitric acid solution and analysed by using quadrupole-induced coupled plasma mass spectrometry (ICP-MS: iCAP Qc, Thermo Fisher Scientific, Bremen, Germany). The Pt reading was normalised to the DNA concentration. For measuring whole cellular Pt, cell lysates were pooled after addition of an equal volume of nitric acid and were digested by using the same method of the Pt–DNA-adduct analysis. The Pt reading in the cells was normalised to the protein concentration.

### Immunofluorescence and immunohistochemistry

For immunofluorescence staining, UMUC3 cells were seeded on an eight-well chamber slide, pre-treated with or without 10 μM DSF and GC (CDDP, 100 µM; GEM, 10 nM) for 1 h and then subjected to immunofluorescence staining by using the primary antibody to ATP7A (Santa Cruz, sc-376467) and the secondary ALEXA Fluor™ 488-conjugated antibody (Abcam, ab150105). Slides were mounted in VECTASHIELD with DAPI (Vector Laboratories, Burlingame, CA, USA). Confocal microscopy images were taken by a Leica TCS SP2, and seven randomly selected fields were quantitatively analysed on Image J.

For immunohistochemical evaluation of ATP7A expression, we used surgical specimens from 31 patients with bladder UC (Supplementary Table 1). All patients had clinically muscle-invasive disease (≥cT2) except for one patient (cT1). All patients received transurethral resection (TUR) of untreated tumour, neoadjuvant CDDP-based systemic chemotherapy (GC in 18, MVAC in 11 and others in 2) and radical cystectomy (RC) at Kyoto University Hospital. After approval by the Institutional Review Board at Kyoto University Graduate School of Medicine (#G0052-17), TUR and RC specimens were subjected to IHC. A trained pathologist (Y. Te.) and two authors (Y.K. and T.K.) independently scored ATP7A staining intensity into four grades (G0–3), and discrepant cases were resolved by discussion with all observers.

### Preparation and characterisation of DSF nanoparticles (DSF-NP)

DSF-loaded nanoparticles were prepared by using an emulsion-solvent evaporation method. Poly (L-lactic acid) (weight–average molecular weight = 5000) (100 mg) and DSF (100 mg) were dissolved in 2 mL of dichloromethane and mixed with 20 mL of 2 wt% polyvinyl alcohol (weight–average molecular weight = 44,000, degree of saponification = 85.5%) aqueous solution. This mixture was homogenised for 30 s by a vortex mixer and sonicated for 10 min to produce an oil-in-water emulsion. The organic phase was evaporated for 12 h at room temperature. The solution was centrifuged at 10,700*g* for 30 min, and the supernatant was centrifuged again at 96,600*g* for 30 min to obtain DSF-incorporated nanoparticles (DSF-NP). DSF-NP was washed twice with water and freeze-dried. The apparent size of NPs was determined by dynamic light scattering by using a ZETASIZER NANO ZS90 (Malvern Panalytical, Almelo, The Netherlands). The amount of DSF entrapped in nanoparticles was determined by using HPLC by UV detection at 250 nm (SHIMADZU LC-20). After dissolution in dichloromethane and evaporation of dichloromethane at room temperature, DSF was dissolved in pure ethanol. The mobile phase was a mixture of acetonitrile:water (80:20), and the flow rate was set at 1 ml/min. Separation was achieved by using a Cosmosil 5C_18_-MS-2 column (150 mm × 4.6 mm).

### Cell-derived xenograft (CDX) model

BALB/cAJcl-nu/nu mice (7-weeks old) were purchased from CLEA Japan Inc. All animal experiments were conducted in accordance with the guidelines of the Laboratory Protocol of Animal Care and Use Committee, Kyoto University. To evaluate tolerability of the combination regimen, three mice were treated with CDDP (4 mg/kg i.v.) and DSF-NP (3 mg/body i.v.). Body weights were measured once a week, and serum creatinine levels were measured 1 week after administration. A UMUC3 subcutaneous xenograft model was prepared by inoculating the right flank of mice with 3.0 × 10^6^ UMUC3 cells with Matrigel®. One week later, the mice were randomly divided into four groups (*n* = 7), receiving saline, CDDP (4 mg/kg body weight), DSF-NP (150 mg/kg body) or CDDP + DSF-NP via tail-vein injection. A T24 subcutaneous xenograft model was prepared by inoculating the right flank of mice with 2.0 × 10^6^ T24 cells with Matrigel®. Two weeks later, the mice were randomly divided into four groups (*n* = 3) receiving saline, CDDP (3 mg/kg body weight), DSF-NP (150 mg/kg body) or CDDP + DSF-NP via the tail vein. The tumour volume was calculated by the following formula: V = (L × W^2) × 0.5, where L is the largest and W is the orthogonal diameters of the tumour.

### Patient-derived xenograft (PDX) models

We established two lines of PDX from human invasive bladder cancer specimens according to previously reported methods.^9,10^ The developed xenograft was divided into 12 equal parts and passaged to 12 CB-17/Icr-crj SCID mice (Charles River, Yokohama, Japan) (7-week old). After 2 weeks, the mice were randomly divided into four groups (*n* = 3) receiving saline, CDDP (4 mg/kg body weight), DSF-NP (150 mg/kg body) or CDDP + DSF-NP via the tail vein. Tumour volume was measured as described above. In both CDX and PDX models, inoculation of cell lines and tumour pieces, and tail injection of drugs, were performed under inhalation anaesthesia with isoflurane. The dose of cisplatin was based on previous reports,^6,11^ and the dose of DSF-NP was determined as 40 nmol/body weight (g), equivalent to the concentration that showed an effect in vitro. Tumour-bearing mice were killed by using carbon dioxide gas at the end of experiment, or when the tumour size exceeded 2 cm in diameter.

### Pt–DNA-adduct staining and TdT-dependent dUTP–biotin nick end labelling (TUNEL) assay

For Pt–DNA-adduct staining, tumour sections were deparaffinised, antigen recovered, blocked with 1% BSA/PBS for 1 h at room temperature, incubated with a 1:500 dilution of anti-CDDP modified DNA, Rat-Mono (CP9/19) (Novus, NBP2-50165) at 4 °C overnight and then incubated with goat anti-Rat IgG (H + L) Cross-Adsorbed Secondary Antibody, Alexa Fluor 546 (1:1000, Invitrogen, A11081). Apoptosis was detected by TUNEL assay by using an apoptosis detection kit (Promega, Madison, WI, USA) following the manufacturer’s instructions.

### Chemosensitivity assay with cancer tissue-originated spheroid (CTOS)

We established CTOSs from PDX and TUR specimens (Table 1), as described.^12^ CTOSs were injected into Matrigel® droplets and cultured in conditioned medium with vehicle, DSF (10 μM), CDDP (10–1000 μM) or DSF + CDDP in 96-well plates. Photographs of each CTOS were taken at days 1 and 6, and the CTOS volume was calculated as follows: volume = (L × W^2) × 0.5, where L is the largest and W is the orthogonal diameters of the spheroid. The growth rate of CTOS was calculated by dividing the volume measured on day 6 by that measured on day 1. A CTOS was defined collapsed if it darkened, and its spherical shape was broken with the cells condensed and dispersed.

**Table 1:** Clinicopathological characteristics and outcomes of 15 urothelial carcinoma patients subject to CTOS assay

Patient #	Age, sex	Pathology, disease stage	Establishment of CTOS (>20 spheres)	Sensivity
#01	83, M	HG, >pT2	No	–
#02	82, M	LG (G1), pTa	Yes	No
#03	75, F	HG with squamous dif., pTa	No	–
#04	69, M	HG (G2 > 3), pT1	No	–
#05	76, M	HG (G2), pTa	Yes	CDDP + DSF
#06	76, M	HG (G2), pTa	No	–
#07	81, M	HG (G3), >pT2	No	–
#08	81, F	HG (G3), >pT1	No	–
#09	47, M	HG (G3), pT2	Yes	CDDP + DSF
#10	49, M	HG (G3), >pT1	No	–
#11	92, M	HG (G2 > G3), pT1	No	–
#12	74, M	HG (G3), pT1	Yes	CDDP alone, CDDP + DSF
#13	87, M	HG, pT1	No	–
#14	69, M	HG (G2), pTa	Yes	CDDP + DSF
#15	80, M	HG (G2), pT1	Yes	CDDP + DSF

*HG* high grade, *LG* low grade, *CDDP* cisplatin, *DSF* disulfiram

### Statistical analyses

All experiments were conducted in triplicate unless otherwise specified. All mean values ± SD reported in the Results section were compared by Student's *t* test. Bonferroni correction was applied to correct for multiple testing. Immunostaining of ATP7A in bladder cancers before and after neoadjuvant chemotherapy was compared by using Wilcoxon ranked-sign test.

The combination index was calculated to evaluate the synergism according to the method reported elsewhere.^13^ Briefly, the derived combination index equation for two drugs is CI = (D)1/(Dx)1 + (D)2/(Dx)2, where (Dx) 1, (Dx) 2 = the concentration of the tested substance 1 and the tested substance 2 used in the single treatment that was required to decrease the cell number by x%, and (D) 1, (D) 2 = the concentration of the tested substance 1 in combination with the concentration of the tested substance 2 that together decreased the cell number by x%. The CI value quantitatively defines synergism (CI < 1), additive effect (CI = 1) and antagonism (CI > 1). All tests were two-sided, and *P-*values < 0.05 were considered statistically significant.

## Results

### High-throughput screening identified disulfiram as a synergic agent with CDDP

We used a chemical compound screen approach to explore the potential synergistic interactions of GC with other compounds in human bladder cancer cells. A library of 2098 known compounds was administered alone or combined with IC_20_ concentrations of GC in UMUC3 and J82 human bladder cancer cells; cell viability was determined after 72 h by using WST-8 assay (Fig. 1a).

We first screened compounds that exhibited sensitisation of GC defined as^1^ enhanced inhibitory effect in combination with GC by 50% or more compared with GC alone and^2^ harmless inhibitory effect in sole administration by 30% or less compared with no treatment. The latter criterion was included to exclude those harbouring a strong antitumour effect by themselves since our primary purpose was to identify compounds that sensitise UC cells to chemotherapy. Initial screening identified 21 compounds for UMUC3 (Fig. 1b) and 47 compounds for J82 cells (Fig. 1c). Eight compounds were commonly identified in both (Fig. 1d, Table 2; Supplementary Table 2). Among those eight, disulfiram (DSF), an FDA-approved drug for alcoholism, demonstrated the most reproducible sensitising effect in the secondary screening as shown in Fig. 1e; we selected DSF for further investigation.

**Table 2:** List of compounds identified in the screening in UMUC3 and J82 human bladder cancer cells

Compound	UMUC3		J82	
	CP alone	GC(IC_20_) + CP	CP alone	GC(IC_20_) + CP
Bonaphton	0.906	0.074	1.044	0.453
Clioquinol	0.999	0.264	0.85	0.172
Demeclocycline hydrochloride	0.87	0.218	0.962	0.367
Disulfiram	0.99	0.144	1.194	0.464
Fludarabine phosphate	1.005	0.099	0.718	0.088
Furazolidone	1.071	0.393	1.238	0.463
Sulconazole nitrate	1.009	0.323	0.918	0.338
Tyloxapol	0.885	0.203	0.846	0.413

*CP* compound, *GC* gemcitabine + cisplatin

Data for CP alone indicate the ratio of OD_450_ for CP alone to OD_450_ for vehicle

Data for GC (IC_20_) + CP indicate the ratio of OD_450_ for GC + CP to OD_450_ for GC

Dose–response assays confirmed that DSF enhanced the inhibitory effects of GC in UMUC3 and J82 cells, with similar effects in three other human UC lines (Fig. 1e). To clarify whether DSF sensitises GEM or CDDP, drug interactions were quantified by median-dose-effect analysis. Combination index assay showed synergistic effects of DSF with CDDP but not with GEM in J82, UMUC3 and T24 cells (Fig. 1f).

### Co-administration of DSF enhances apoptosis by GC through reactive oxygen species (ROS) accumulation

**Fig. 2:**
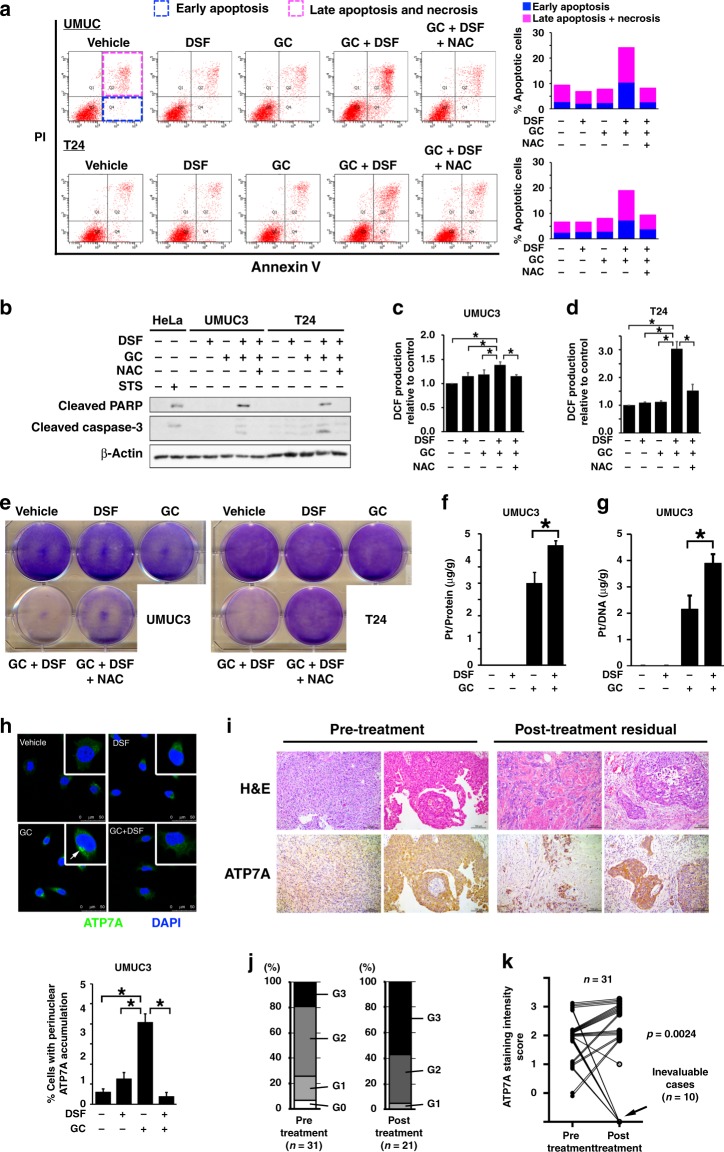
Co-administration of DSF increases DNA–Pt adducts and ROS production, and enhanced apoptosis with perinuclear localisation of cisplatin efflux transporter ATP7A precluded. **a** Apoptosis was evaluated with Annexin V-FITC/propidium iodide and flow cytometry in cells treated as indicated for 36 h. The effects of the antioxidant (NAC) were also evaluated. **b** Western blot analysis for cleaved PARP and cleaved caspase 3 levels in UMUC3 and T24 cells treated as indicated for 36 h. HeLa cells were treated with 1 μM of staurosporine (STS), which is known to induce apoptosis. **c**, **d** ROS production was assessed by DCF. UMUC3 (**c**) and T24 (**d**) cells were treated as indicated for 18 h. The effect of NAC was also evaluated. *p < 0.05. **e** Colony-formation assays were performed in UMUC3 and T24 cells treated as indicated with addition of NAC (1 mM) for 72 h. Cells were stained with crystal violet. **f** Whole-cell Pt accumulation. UMUC3 cells were treated with 10 μM DSF and GC for 1 h, and then whole-cell lysates were subject to ICP-MS assay to quantify intracellular Pt accumulation (normalised by cellular protein amount). *p < 0.05. **g** Pt–DNA-adduct accumulation assessed by ICP-MS. UMUC3 cells were treated with 10 μM DSF and GC for 1 h, and cellular DNA was extracted and subjected to ICP-MS assay (normalised by DNA amount). *p < 0.05. **h** Top: confocal microscopy images showing the effect of GC and DSF on the intracellular localisation of ATP7A (green fluorescence). UMUC3 cells were treated as indicated for 1 h. Nuclei were stained with DAPI (blue). Bars indicate 50 μm. Bottom: quantitative analysis of the percentage of cells with perinuclear accumulation of ATP7A. Seven randomly selected microscopic fields were quantitatively analysed by Image J. **p < 0.01. **i** Representative microphotographs of pre-treatment and post-neoadjuvant chemotherapy (post-chemotherapy) bladder urothelial carcinoma by using H&E and ATP7A staining. Bars indicate 100 μm. **j** Distribution of ATP7A staining intensity score for pre-treatment (*n* = 31) and post-chemotherapy (*n* = 21) tumours. Post-chemotherapy tumours could not be evaluated in ten cases with no or very little viable tumour lesions (pT0 in 4, pTis in 4, pT1 in 1 and pT3b in 1). **k** Statistically significant changes in ATP7A staining intensity score between pre-treatment and post-chemotherapy tumours in the 31 patients (Wilcoxon’s ranked-sign test was applied for 21 patients with post-chemotherapy residual tumour)

CDDP cross-links to DNA, while GEM is incorporated into DNA strands, and consequently both induce apoptosis via blockade of DNA replication and transcription.^14,15^ Oxidative damage has also been implicated in the cytotoxic effect of CDDP.^16^ To determine the mechanism by which DSF enhances the inhibitory effects of GC, we first examined whether DSF combined with GC enhances apoptosis in UMUC3 and T24 cells. Both early and late apoptotic cell populations were increased by GC in combination with DSF (GC + DSF) compared with GC alone (Fig. 2a). Increased expressions of cleaved PARP and caspase 3 in cells treated with GC + DSF (Fig. 2b) suggested that the apoptosis induced by GC + DSF depended on the caspase pathway.

We next assessed the effect of co-administration of DSF on intracellular redox status by quantitative analysis of intracellular ROS. Both UMUC3 and T24 cells treated with GC + DSF showed significant increases in DCF production after DCFH-DA treatment, suggesting increased ROS production (*p* < 0.05; Fig. 2c, d). The role of increased ROS production in DSF-induced sensitisation to CDDP was strongly supported by our observations that the antioxidant N-acetyl-L-cysteine (NAC), which effectively blocked DCFH-DA-induced ROS production (Fig. 2c, d), inhibited the induction of apoptosis (Fig. 2a) and cleaved PARP and caspase 3 expressions (Fig. 2b). NAC also partly reversed the inhibition of colony-forming ability in cells treated with GC + DSF (Fig. 2e). These results indicate that co-administration of DSF enhances apoptosis induced by GC, which is partly attributed to ROS accumulation.

### Co-administration of DSF alters ATP7A localisation and increases intracellular accumulation of CDDP and Pt–DNA adducts

Cells have been reported to exert resistance to CDDP by accelerating efflux or blocking influx of CDDP.^17–21^ To investigate the effect of DSF on intracellular CDDP accumulation, we quantified intracellular Pt after CDDP treatment in the absence or presence of DSF by using ICP-MS. Intracellular Pt significantly increased in cells treated with GC + DSF compared with GC alone (*p* < 0.05; Fig. 2f). Combination therapy also doubled Pt–DNA adducts (Fig. 2g), a more direct evidence of cross-link between CDDP and DNA that interferes with DNA replication and subsequent mitosis.

Multiple membrane transporters, such as MDR1, MRP1 and ABCG2, have been reported to be associated with drug resistance. Among them, we focused on copper transporters, including CTR1, CTR2, ATP7A and ATP7B, since they are known to incorporate or eliminate Pt.^15^ Of these, the expression of ATP7A was decreased by DSF treatment, while the other transporters showed no significant changes (data not shown). The functions of some membrane transporters are regulated by subcellular localisation.^19,20,22,23^ We thus examined the effect of DSF on subcellular localisation of ATP7A, a copper efflux transporter, by using immunofluorescent microscopy (Fig. 2h). ATP7A was recruited to perinuclear *trans*-Golgi upon GC treatment, as reported previously.^20,23^ This perinuclear accumulation is required for ATP7A to function as a Pt efflux transporter. Interestingly, significantly fewer cells showed perinuclear accumulation of ATP7A upon GC + DSF treatment, suggesting that DSF increases intracellular CDDP through inhibiting subcellular localisation and efflux function of ATP7A. These findings suggest that DSF confers sensitivity to CDDP to human UC cells through inhibiting the efflux function of the ATP7A Pt transporter, resulting in increased Pt–DNA adducts, ROS production and cellular apoptosis.

### Clinical relevance of ATP7A to sensitivity and resistance to CDDP in human bladder UC

We next examined the clinical relevance of ATP7A in UC patients receiving CDDP-based chemotherapy. We examined ATP7A expression by using IHC on paired bladder UC specimens before and after CDDP-based chemotherapy. We identified 31 patients who received TUR for untreated tumour and neoadjuvant CDDP-based systemic chemotherapy before RC at our hospital to use matched surgical specimens from TUR pre chemotherapy and RC post chemotherapy. All patients had muscle-invasive bladder UC with a single exception of T1 high-grade tumour. Moderate-to-strong ATP7A expression (G2 or G3) was observed in over 70% of pre-treatment tumours (Fig. 2i, left and 2j, left), although we could not evaluate subcellular localisation of ATP7A by using IHC unfortunately. Consistent with previously reported outcomes,^24^ approximately one-third (*n* = 10) of patients showed marked pathological response, but irrespective of ATP7A expression in pre-treatment tumour tissue (Fig. 2k). ATP7A expression in post-NAC tumours could not be evaluated in the ten patients due to no or little residual tumour lesion. Of the remaining 21 patients with viable residual tumour lesions in RC specimen, 95% (20 of 21) showed moderate-to-strong ATP7A expression (Fig. 2j, right and 2k). Although the results of our in vitro experiments indicated that expression levels of ATP7A do not necessarily reflect its activity, we found that ATP7A is at least expressed in most muscle-invasive bladder UC, particularly in cases resistant to CDDP-based treatment, suggesting the potential for ATP7A as a target in the treatment of chemoresistance.

### DSF-NP enhances CDDP effects in vivo

**Fig. 3:**
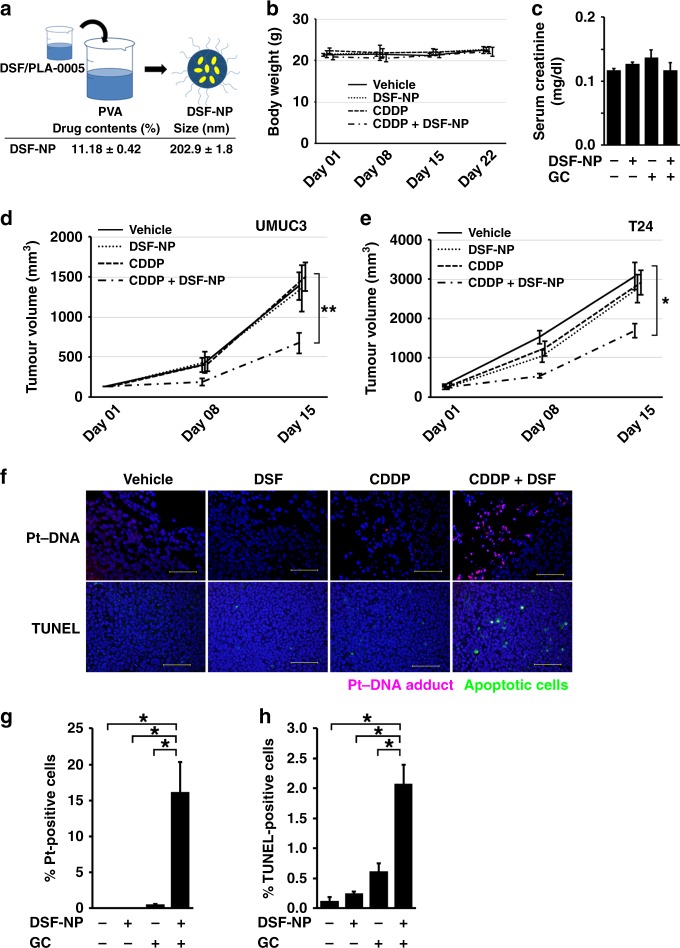
DSF–polylactic acid (PLA) nanoparticles (DSF-NP) enhance the inhibitory effect of cisplatin treatment in mice. **a** DSF micellar PLA nanoparticles (NP) were prepared via emulsion-solvent diffusion method by using polyvinyl alcohol (PVA). **b**, **c** Tolerability of the combination regimen. Seven-week-old female athymic mice (*n* = 3) were treated with CDDP (4 mg/kg i.v.) and/or DSF-NP (3 mg/body i.v.). Body weights were measured once a week (**b**), and serum creatinine levels were measured 1 week after administration (**c**). **d**, **e** Tumour growth rate in the subcutaneous UMUC3 (**d**, *n* = 5 for each group) or T24 xenograft models (**e**, *n* = 3 for each group). Tumour-bearing mice were treated with vehicle, DSF-NP (3 mg/body i.v.) alone, CDDP (4 mg/kg i.v. for UMUC3 and 3 mg/kg i.v. for T24) alone or CDDP in combination with DSF-NP. *p < 0.05, **p < 0.01 (CDDP + DSF-NP vs. single treatment). **f** Formation of Pt–DNA adducts (top row, red) and apoptosis (bottom row, green) in tumour cells of UMUC3 xenografts as in **d** detected by anti-Pt–DNA-adduct antibody (day 1) and TUNEL assay (day 15), respectively. Bars indicate 100 μm. **g**, **h** Quantitative analysis of Pt–DNA-positive cells (**g**) and TUNEL-positive cells (**h**) in tissue sections from tumours shown in **f**. Three randomly selected microscopic fields were quantitatively analysed with Image J. *p < 0.05

We next evaluated the efficacy and anticancer effects of CDDP with DSF in vivo. DSF is quickly degraded in the gastrointestinal system, during the hepatic first-pass effect, and in the bloodstream.^25^ Indeed, in our pilot experiments, oral administration of the native form of DSF showed little or very modest effects in sensitising mouse tumours to CDDP (data not shown). To deliver DSF to tumours in an unmetabolised form in vivo, micellar DSF-NP was prepared by emulsion-solvent diffusion method (Fig. 3a). DSF-NP was suspended in physiological saline and administered by tail-vein injection. Co-administration of CDDP + DSF-NP to non-tumour-bearing mice showed no adverse effects on body weight (Fig. 3b) and renal function (Fig. 3c).

We investigated the efficacy of DSF-NP + CDDP by using cell-based subcutaneous xenograft models. Tumour-bearing mice were treated with DSF-NP and CDDP at doses determined in advance that individually did not significantly inhibit tumour growth. Compared with CDDP alone, the combination treatment significantly inhibited tumour growth by 45% in UMUC3 (*p* < 0.01; Fig. 3d) and by 40% in T24 xenograft models (*p* < 0.01; Fig. 3e). Tumours treated with DSF-NP + CDDP also exhibited increased Pt–DNA adducts (Fig. 3f, top and Fig. 3g) and apoptosis (Fig. 3f, bottom and Fig. 3h), consistent with our in vitro results. Collectively, these results suggest that DSF-NP effectively sensitised cell-based xenograft tumours to CDDP in vivo via increasing intracellular accumulation of CDDP and consequent apoptosis.

### Patient-derived preclinical models for predicting the benefit of DSF co-administration with CDDP

We showed that the majority of muscle-invasive bladder UC harboured resistant cells within the tumour with ATP7A upregulation. In contrast, a subset of patients responded very well to conventional CDDP-based chemotherapy without modification. These findings indicate the need for pre-treatment prediction of clinical efficacy of CDDP-based chemotherapy to identify patients who will benefit from additional DSF. We thus next tried to model a prediction system.

**Fig. 4:**
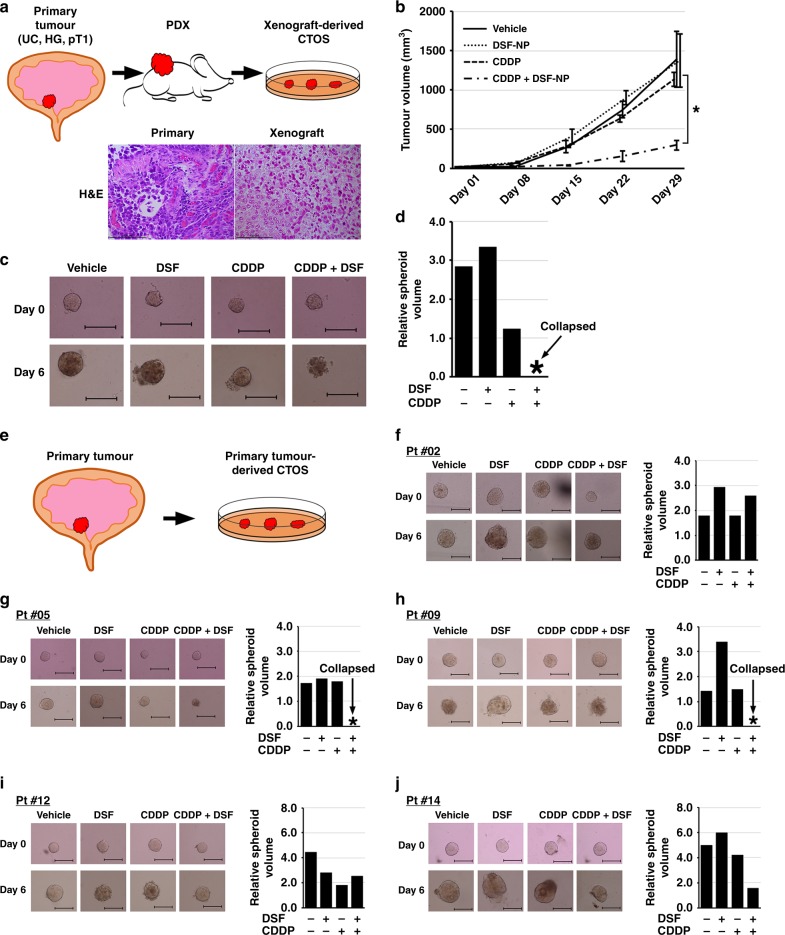
DSF enhances the inhibitory effect of CDDP in preclinical models. **a** Schematic diagram of PDX and xenograft-derived CTOS model. Subcutaneous PDX lines were established from transurethrally resected specimens of primary tumour (high-grade urothelial carcinoma, pT1; HG, UC and pT1), and PDX tumours were subject to CTOS culture. H&E microphotographs show histological similarity between primary and xenograft tumours. Bars represent 100 μm. **b** Tumour growth rate in the subcutaneous PDX models. Two weeks after implantation of tumours, mice were randomised into four groups (*n* = 3) and treated with vehicle, DSF-NP (3 mg/body i.v.), CDDP (4 mg/kg i.v.) or CDDP in combination with DSF-NP. *p < 0.05 (CDDP + DSF-NP vs. single treatment). **c** Representative images of xenograft-derived CTOS in pre- (day 0) and post-treatment (day 6) settings. Note that CTOS treated with CDDP and DSF collapsed, while others grew and maintained a spheroid shape. **d** Relative volumes of spheroids on day 6 to those on day 0. **e** Schematic diagram of the direct primary tumour-derived CTOS model. Transurethrally resected specimens of primary tumours were directly subject to CTOS culture. **f**–**k** Representative images of CTOS derived from primary tumours from five patients pre- (day 0) and post treatment (day 6) (left), and relative volumes of spheroids on day 6 to those on day 0 (right)

We and others have reported the utility of other patient-derived model systems, namely PDX,^10,26^ organoid culture^26^ or cancer tissue-originated spheroid (CTOS).^12,27^ To determine whether CTOS, which shows advantages in generating rapid results, can predict treatment efficacy in vivo, we first evaluated the efficacy of the combined treatment in PDX and CTOS (Fig. 4a). Bladder cancer (high-grade UC, pT1) tissues obtained by TUR were subcutaneously implanted in the flank of SCID mice and established as a xenograft line that can be stably passaged up to at least ten times.^10^ These PDX lines preserved histological characteristics of high-grade UC (Fig. 4a), as reported previously.^10,26,28^ We also established CTOS from the PDX (PDX-derived CTOS), according to the previously reported method.^12^ DSF combined with CDDP significantly inhibited PDX tumour growth (*p* < 0.05), while CDDP or DSF-NP alone did not show significant efficacy (Fig. 4b). The combinatorial treatment showed marked efficacy, as demonstrated by shrunk or collapsed spheroids on day 6 (Fig. 4c, d). We observed another example from a different case with high-grade UC, in which we did not observe enhanced inhibitory effect of the combined treatment in PDX or CTOS systems (Supplementary Fig. 1). These results suggest a correlation between CTOS and PDX systems with regard to response to the DSF + CDDP combination treatment.

We next evaluated the patient-derived CTOS culture as drug susceptibility system (Fig. 4e). We successfully obtained sufficient numbers of spheroids for the test in 6 (40%) of 15 patients with bladder UC (Table 1). Of those six, co-administration of CDDP and DSF showed efficacy in CTOS from four cases (#5, #9, #14 and #15; Fig. 4g, h, j, k, respectively), while one responded to CDDP alone (#12; Fig. 4i) and one did not respond to either treatment (#2; Fig. 4f).

If clinical decisions were made based on the CTOS results, cases #5, #9, #14 and #15 would have been recommended CDDP and DSF combination therapy, while case #12 would have been proposed conventional CDDP-based chemotherapy, and case #2 would have been proposed other chemotherapy or immune checkpoint inhibitors. Unfortunately, only one case (#9) received CDDP-based chemotherapy, making it difficult to confirm the correlation between CTOS assay results and real clinical response. Nonetheless, case #9 showed clinical response to GC chemotherapy, which correlates with the CTOS results. This patient underwent three courses of neoadjuvant GC followed by radical cystectomy for high-grade, pT3N1M0 bladder UC (Supplementary Fig. 2). GC chemotherapy yielded insufficient radiological and pathological response, as would have been predicted from the CTOS assay. Although very preliminary, these findings indicate the potential for the CTOS system as a promising tool for the quick prediction of treatment benefit from DSF combined with CDDP.

## Discussion

We identified DSF as a CDDP sensitiser by using high-throughput chemical screening from a library of known compounds. Despite the development of immune checkpoint inhibitors in cancer treatment, these treatments show limitations in practice. Therefore, CDDP-based chemotherapy remains the mainstay treatment for unresectable UC, and how to maximise the benefit from CDDP-based chemotherapy for UC as well as other cancers is a critical question. Our results thus have a strong impact on cancer chemotherapy in general.

Although the initial radiological response rate is relatively high in metastatic UC patients who receive first-line CDDP-based chemotherapy, intrinsic resistance accounts for ~30% of metastatic UC.^4^ In a neoadjuvant setting with standard dose regimens, the pathologic complete response (CR) (pT0) rate is only 10–15%,^24^ while the CR rate is ~40% in recent studies with dose-dense MVAC protocol.^29^ Indeed, the CR rate was 13% in our neoadjuvant chemotherapy series, and ten patients had marked pathological regression by the systemic chemotherapy. ATP7A expression in pre-treatment tumour tissue was not associated with excellent pathological response. These tumours might have somatic mutations in *ERCC2*^30^ or DNA damage response genes, such as *ATM*, *FANCC* and *RB1*,^31^ which sensitise tumour cells to CDDP irrespective of ATP7A activity, although we did not test them in the present study. However, the remaining patients with resistant tumour lesion showed robust ATP7A expression, suggesting its association with chemoresistance. Therefore, DSF combined with CDDP-based chemotherapy is primarily expected to improve initial response in primary chemotherapy for metastatic UC or preoperative neoadjuvant chemotherapy for localised or locally advanced diseases. This is particularly important as dose-dense MVAC^29^ or GC^32^ has shown promising results with a higher rate of radiological or pathological CR than conventional chemotherapy with the standard dose. These findings indicate that improvement in drug delivery is relevant to UC, and we can expect better response to CDDP-based chemotherapy combined with DSF. This also highlights the importance for efficacy prediction, such as CTOS models. Sensitive tumours to cisplatin may only require standard chemotherapy, whereas other patients would benefit from DSF co-administration with CDDP-based chemotherapy. Our findings demonstrate the potential usefulness of CTOS models as a tool for drug efficacy.

In the present study, we identified DSF by using a drug repurposing strategy. DSF has been used for over six decades as a treatment for alcoholism, with well-established pharmacokinetics, safety and tolerance.^33^ Indeed, our preclinical studies showed no severe adverse effects in mice, given clinically applied doses of DSF and CDDP. Moreover, we identified very few reports on adverse events for DSF with CDDP and other Pt agents in a public database such as the US Food and Drug Administration Adverse Event Reporting System (FAERS, https://www.fda.gov/drugs/guidancecomplianceregulatoryinformation/surveillance/adversedrugeffects/ucm070093.htm) and the Japanese Adverse Drug Event Report (JADER, https://www.pmda.go.jp/safety/info-services/drugs/adr-info/suspected-adr/0006.html). Thus, repurposing of DSF to a chemotherapy sensitiser is a promising treatment strategy supported by sufficient regulatory information.

DSF has been a previous candidate for drug repurposing in oncology. Several preclinical studies showed that DSF has anticancer activity.^34–39^ Sodium dithiocarb, a DSF metabolite, was used to treat high-risk human breast cancer in a clinical trial.^40^ DSF also enhanced the anticancer efficacy of chemotherapeutic agents and was effective as a single agent.^41^ Other mechanisms for the anticancer activity of DSF have been reported. DSF inhibits proteasome activity.^42^ DSF also chelates bivalent metals and forms complexes with copper, which enhances its antitumour activity.^43^ A recent epidemiological study supports these findings; patients who continuously used DSF had a lower risk of death from cancer compared with those who stopped using DSF at their diagnosis,^39^ suggesting that metabolites of DSF also have some antitumour effects. DSF also exhibits tumour-suppressing effects by blocking NPL4. Thus, anticancer effects of DSF have long been known. However, it is noteworthy that the present study is the first report identifying DSF as a CDDP sensitiser via an unbiased, systematic approach of chemical library screening.

Here we demonstrated for the first time that DSF acts as a CDDP sensitiser in vivo by using a PDX model. The unmodified form of DSF is extremely unstable.^25^ Indeed, little sensitising activity was observed with oral administration of DSF in our model. Nanocarriers have recently attracted attention as antitumour drug delivery systems,^44,45^ and other researchers have also generated DSF nanoparticles.^11,46,47^ Although the preparation methods need improvement, we demonstrated that DSF-NP can enhance intratumoural accumulation of Pt–DNA adducts and apoptosis. We also expect that enhanced permeability and retention (EPR)^48^ can strengthen the cancer-specific efficacy of DSF. Moreover, nano-formulation is also promising in allowing labelling for specific uptake by cancer cells.^49^ On the other hand, DSF-NP has never been used in human, which may weaken the advantage of drug repurposing. Careful safety assessment will be needed before clinical use of DSF-NP.

We also report a previously unknown association between DSF and ATP7A in chemoresistance. We demonstrated that DSF increased intracellular accumulation of Pt–DNA adducts and sensitisation to CDDP. ATP7A is an important transporter for copper and Pt, and its congenital dysfunction causes Menkes disease.^19,22,23,50^ Importantly, subcellular localisation of ATP7A is a determinant for its transporter activity,^23,50^ which may be why previous studies by using gene expression analysis failed to identify the involvement of ATP7A in chemoresistance. We found that ATP7A was recruited to Golgi body in response to CDDP in the absence of DSF, and co-administration of DSF prevented this subcellular re-localisation.

Based on the results, it is expected that co-administration of DSF-NP yields an antitumour efficacy of low-dose CDDP equivalent to that obtained at the standard doses. This indicates that DSF-NP may be useful for CDDP-ineligible patients who have impaired renal function, which limits the dose of CDDP. Co-administration of DSF-NP may provide benefit from CDDP at a tolerable dose. On the other hand, one concern is that DSF-NP may increase the risk of CDDP-induced nephrotoxicity, since the cancer-specific mechanism of action of DSF-NP has not been clearly determined. In this regard, however, our in vivo pilot study showed that co-administration of DSF-NP did not induce renal dysfunction as assessed by serum creatinine. Furthermore, our in vivo pilot studies showed no sign of significant adverse effects, including gastrointestinal and haematological toxicities.

We also believe that co-administration of DSF-NP with CDDP is worth exploring for patients showing acquired resistance after initial response to CDDP-based chemotherapy. Recent publications have attributed acquired chemoresistance to cellular hierarchy with differential chemosensitivities, which leads to repopulation of tumours with resistant cells.^51,52^ Therefore, improvement in chemosensitivity by DSF-NP seems relevant even to the acquired resistance, although the functional significance of ATP7A with regard to each hierarchical population in UC should be studied in the future.

Recently, the usefulness of 3D cell cultures, such as organoid^26^ and CTOS,^12^ has attracted attention in cancer research. Lee et al.^26^ showed that organoids of bladder cancer inherited the biological properties of clinical specimens, and can be used for drug screening. We confirmed that drug susceptibility by using our CTOS system was parallel with PDX, indicating that it is a promising tool to predict whether combination treatment will be effective or not. These 3D cell culture systems show important advantages in quick establishment, low cost and technical feasibility for drug susceptibility assessment.

The present study has several limitations. The initial screening strategy was aimed to identify compounds that sensitise GC chemotherapy; however, later analyses showed that DSF sensitises tumour cells to CDDP but not GEM. Therefore, it is not clear whether GEM has any role in this context. We only partially clarified the mechanism of action for DSF sensitising cancer cells to CDDP. Particularly, we did not address the link between Pt–DNA adducts and ROS production, which has not been fully elucidated and seems to be beyond the scope of the present study. In vivo models used in the present study were based on subcutaneous tumour, but not orthotopic. Although we demonstrated potential clinical application for efficacy prediction by using a patient-derived CTOS system, the number of cases was limited, and they included non-muscle-invasive diseases. Since the establishment of a sufficient number of CTOS from muscle-invasive bladder cancer is relatively difficult at present, we conducted CTOS assays including non-muscle-invasive bladder cancer in the study. In addition, many of the CTOS assays in the study were done as a single experiment. With future modification of the CTOS system, those experiments can be done in multiplicate. Overcoming this will make it possible to conduct sensitivity prediction tests that more closely match the clinical needs. Nonetheless, we present a successful example for a novel strategy to enhance the efficacy of CDDP-based chemotherapy, from high-throughput chemical screening of repositionable drugs, via functional verification and development of drug susceptibility evaluation systems by using in vitro and in vivo models, to in silico screening for safety assessment prior to clinical application.

## Supplementary information

Supplementary material

## Data Availability

All data and material are available upon request.
