# Supplementary material for: Systematic chemical screening identifies disulfiram as a repurposed drug that enhances sensitivity to cisplatin in bladder cancer: a summary of preclinical studies
Source: Br J Cancer. 2019 Nov 1;121(12):1027–38. doi: 10.1038/s41416-019-0609-0 (PMC6964684; doi:10.1038/s41416-019-0609-0)
Supplement: Supplementary file 1 — Supplementary material [file 41416_2019_609_MOESM1_ESM.docx]

**Supplementary information**

**Systematic chemical screening identifies disulfiram as a repurposed drug that enhances sensitivity to cisplatin in bladder cancer: a summary of preclinical studies**

by Yuki Kita, et al.

Legend for supplementary figure 1

Legend for supplementary figure 2

Supplementary figure 1

Supplementary figure 2

Supplementary table 1

Supplementary table 2

**Legends for supplementary figures**

**Supplementary figure 1**

**A.** Schematic diagram of patient-derived xenograft (PDX) and xenograft-derived cancer tissue originated spheroid (CTOS) model. Subcutaneous PDX lines were established from radical cystectomy specimen of primary tumor (high-grade urothelial carcinoma, pT4) and PDX tumors were subject to CTOS culture. H&E microphotographs show histological similarity between primary and xenograft tumors (Bars represent 100 μM). **B**. Tumor growth rate in the subcutaneous PDX models. Two weeks after implantation of tumors, mice were randomized into four groups (n = 2) and treated with either vehicle, DSF-NP (3 mg/ body i.v.), CDDP (4 mg/kg i.v.), or CDDP in combination with DSF-NP. *p<0.05 (CDDP + DSF-NP vs each of others). **C.** Representative images of xenograft-derived CTOS in pre- (Day 0) and post-treatment (Day 6) settings. **D.** Chart for the relative volumes of spheroids on day 6 to that on day 0.

**Supplementary figure 2**

Radiological and pathological responses of a patient who had originated CTOS#9 and received three course of systemic gemcitabine and CDDP followed by radical cystectomy. Pre-treatment (pre-chemotherapy) contrast-enhanced computed tomography (CECT) shows highly enhanced tumors occupying the bladder and invading peri-vesical fat tissue indicating cT3 disease (left, top). A swollen right obturator lymph node (arrow) is also noted (left, middle). Transurethrally resected specimen showed high-grade urothelial carcinoma cells invading muscle layer (left, bottom). After neoadjuvant chemotherapy, the bladder (right, top) and lymph node (right, middle) lesions have reduced in size only partially, and pathological examination of radical cystectomy specimen revealed degenerated but viable carcinoma cells both in bladder (right, bottom) and the lymph node. Bars indicate 100 μm.

Supplementary figure 1


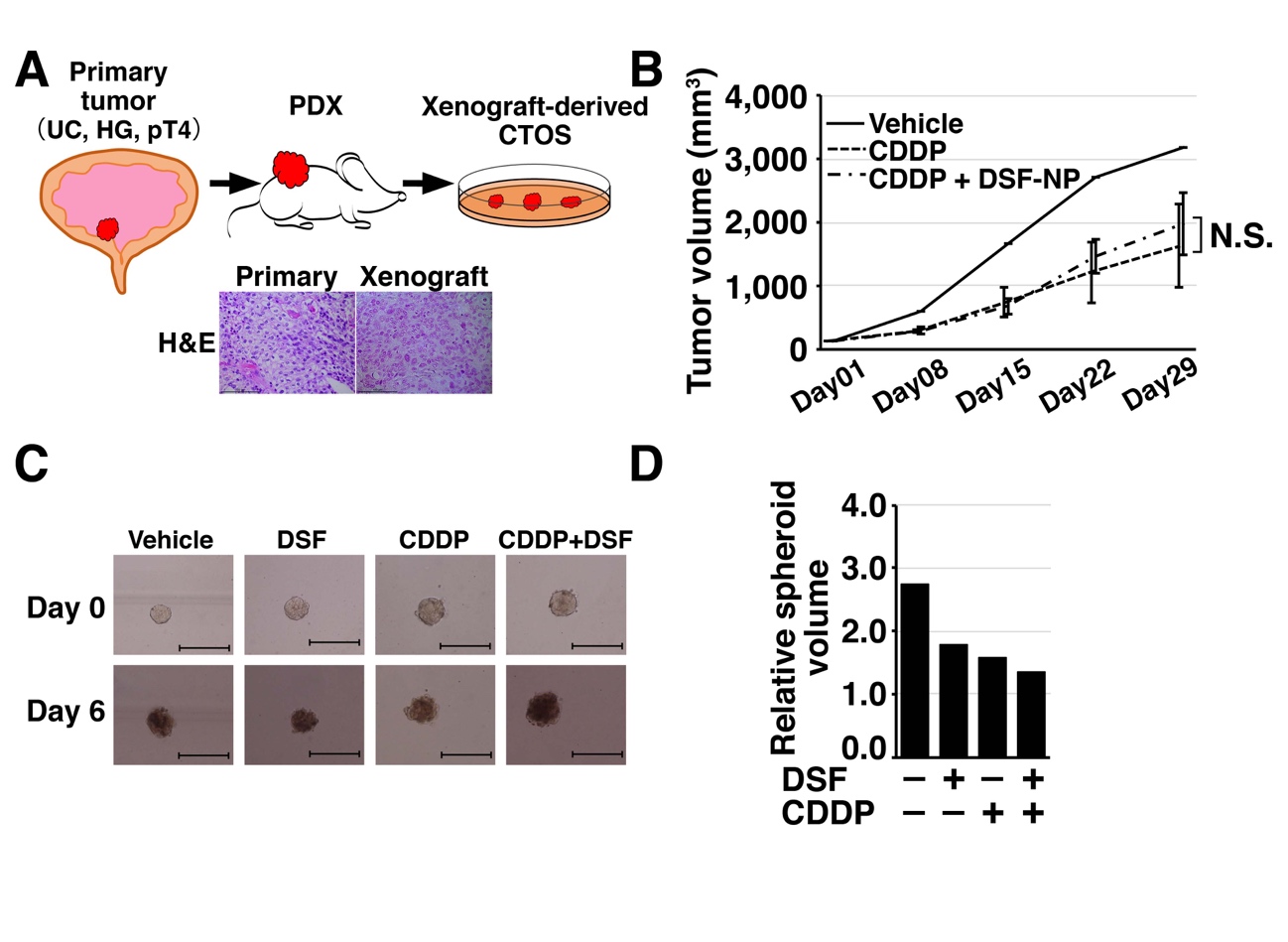


Supplementary figure 2


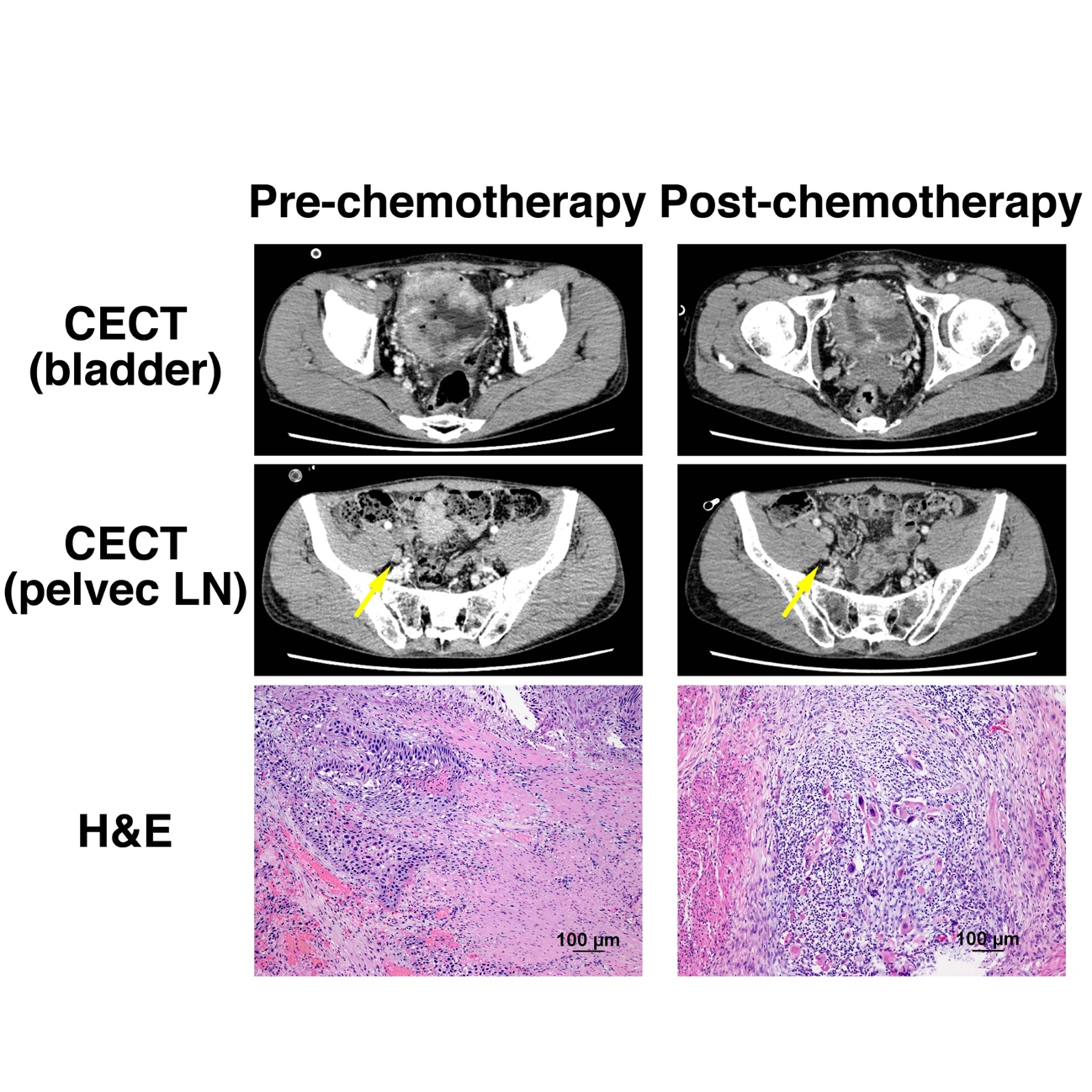


Supplementary Table 1

Clinicopathological characteristics of 31 patients with bladder UC

Supplementary Table 2

Result of initial screening
